# Towards bioinformatics assisted infectious disease control

**DOI:** 10.1186/1471-2105-10-S2-S10

**Published:** 2009-02-05

**Authors:** Vitali Sintchenko, Blanca Gallego, Grace Chung, Enrico Coiera

**Affiliations:** 1 Centre for Health Informatics, University of New South Wales, Sydney, New South Wales, Australia; 2 Centre for Infectious Diseases and Microbiology, Western Clinical School, The University of Sydney, Sydney, New South Wales, Australia

## Abstract

**Background:**

This paper proposes a novel framework for bioinformatics assisted biosurveillance and early warning to address the inefficiencies in traditional surveillance as well as the need for more timely and comprehensive infection monitoring and control. It leverages on breakthroughs in rapid, high-throughput molecular profiling of microorganisms and text mining.

**Results:**

This framework combines the genetic and geographic data of a pathogen to reconstruct its history and to identify the migration routes through which the strains spread regionally and internationally. A pilot study of *Salmonella typhimurium *genotype clustering and temporospatial outbreak analysis demonstrated better discrimination power than traditional phage typing. Half of the outbreaks were detected in the first half of their duration.

**Conclusion:**

The microbial profiling and biosurveillance focused text mining tools can enable integrated infectious disease outbreak detection and response environments based upon bioinformatics knowledge models and measured by outcomes including the accuracy and timeliness of outbreak detection.

## Background

Current early warning systems successfully detect large outbreaks but show significant delays and low sensitivity in detecting moderate and small epidemics [1,2]. It is also estimated that a one-week delay in the implementation of control measures for SARS resulted in a 2.6-fold increase in the mean epidemic size and a four-week extension of the mean epidemic duration [2].

The body of knowledge on the molecular profiles and epidemiology of pathogens accumulated in databases and biomedical literature has been also expanding. We have argued that bioinformatics pathogen profiling can help to predict patient outcomes, identify markers that can be applied for early diagnosis and enable tailored interventions, commonly referred to as "personalized medicine" [3]. We propose in this paper a novel framework for bioinformatics assisted biosurveillance and therapy that addresses the inefficiencies in traditional public health surveillance as well as the need for more timely and comprehensive infectious disease monitoring and control. It leverages from recent breakthroughs in high-throughput molecularprofiling of microorganisms and text mining as well as upon the growing electronic sources of knowledge about the molecular epidemiology of pathogens with epidemic potential.

A pathogen profile can be considered as a single multivariate observation or a set of observations, comprised of classes of specific attributes, which are designed for a comparison with a knowledgebase. In contrast to traditional subtyping based on phenotypic characteristics, such as serotype, biotype, phage type or antibiogram (susceptibility to antimicrobials), genetic profiling describes the phenotypic potential within the nucleic acid sequence. Sequence-based typing on one hand and randomly amplified polymorphic DNA, plasmid fingerprinting or pulsed-field gel electrophoresis (PFGE) on the other, can be viewed as examples of direct and indirect methods of assessing nucleic acid sequence, respectively. They provide both strain typing and key phylogenic data [3].

Methods based on repetitive sequences present at several loci in the genome have been developed with the potential to generate consistent pathogen profiles that are amenable to standardization and database cataloging. Genotyping systems based on comparison of sizes and numbers of different DNA fragments separated by gel electrophoresis express their results in continuous values which are less reliable than direct sequence-based methods, due to the lack of precision and reproducibility. Typing systems that use markers with categorized values (e.g., multilocus sequence typing, MLST) are intrinsically highly reproducible and more appropriate for library typing. Typing techniques like MLST that allow classification consistent with phylogenetic relationships are useful tools for studying global epidemiology. However, techniques that provide real-time or near real-time typing capability are likely to provide the most relevant information and therefore have the most impact.

## Results

### Illustrative scenario

The emergence of multi-resistant *Staphylococcus aureus *(MRSA) as a significant cause of hospital- and community-acquired morbidity and mortality has prompted calls for better surveillance systems supported by analysis of the molecular epidemiology of MRSA [4]. Different genotypes produce many virulence factors associated with certain severe infections and caused by MRSA clones distributed worldwide. Accumulating evidence suggests associations between specific MRSA genotypes, clinical syndromes and patient outcomes. Specifically, super-antigenic exotoxins appear to be major virulence factors in hospital MRSA clones, and enterotoxin A may be involved in the septic shock. Panton Valentine Leucocidin (PV) has emerged as a virulence factor in community-acquired MRSA infections responsible for the high mortality associated with necrotizing pneumonia [5].

**Figure 1:**
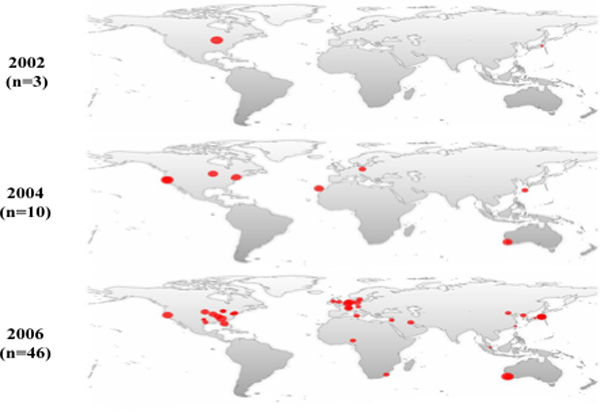
Reports of virulent MRSA SCCmec IV outbreaks in 2002, 2004 and 2006 .

There is an increasing benefit in using rapid molecular profiling for MRSA outbreak detection. For example, rapid outbreak detection by rapid MRSA *spa *typing has become a potential alternative to traditional approaches to hospital-acquired infection control [3,6]. In one study, automated clonal alerts, based on real-time *spa *typing of hospital MRSA isolates and temporal-scan test statistics, were 100% and 95.2% sensitive and specific, respectively, in identifying outbreaks and more sensitive and timely than routine surveillance [6]. The magnitude of the surge in reports and the geographical spread of virulent MRSA clones are illustrated in Figure 1. However, this growing body of retrospective evidence remains underutilized in the cluster investigations. There is an urgent need for tools that can alert clinicians about trajectories of the spread of microbial clones of public health significance.

### Genotyping assisted infectious disease control

**Figure 2:**
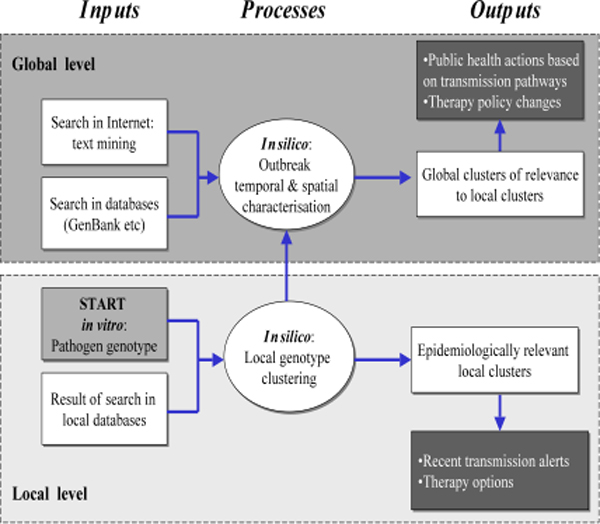
Genotyping assisted infectious disease control framework.

The conceptual framework of bioinformatics assisted infectious disease control presents a case how microbial genomics can be integrated with clinical data to cluster patients belonging to the same outbreak or to predict responses to treatment (Figure 2). Microbial genotype data and search results are inputs and early warnings are outputs in the model. Genotype mining and matching environments utilise biomedical text and database searches for phenotype-to-genotype studies (direct association analysis of candidate genes), genotype-to-phenotype studies and whole genome analysis (indirect association analysis) in order to generate knowledge (i.e., rules and algorithms) concerning the relationships among microbial genes, antimicrobials and patient outcomes, and the effects of gene variation on these relationships. This framework combines the genetic and spatiotemporal data of a pathogen to reconstruct its history and to identify many of the migration routes through which the strains spread regionally and internationally.

The searches and analyses are conducted on two levels. First, genotypes or pathogen profiles are compared with local databases to detect recent transmission or to predict therapy outcomes. Then, global search is performed to collect the information about temporal and spatial distribution of genotypes or profiles obtained, in order to discover transmission pathways and enhance clinical policies. Once a profile is constructed, it can be matched with those of others or with existing datasets using similarity measures and clustering techniques. Probabilistic or information theoretic distance functions and clustering algorithms have been applied to match microbial profiles [3].

### Genomic profiling of pathogens, knowledge representation and text mining

**Figure 3:**
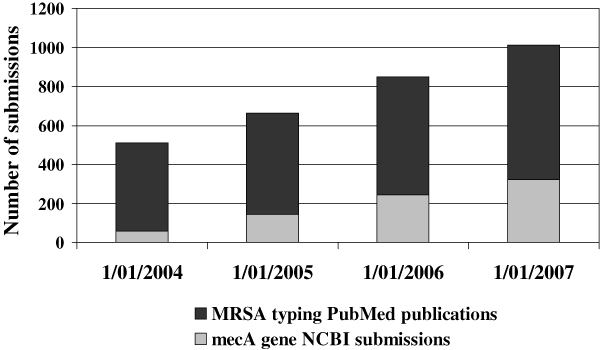
The dynamics of reports on *mecA *gene responsible for methicillin resistance in MRSA.

A pathogen profile is a synthesis of various markers and clinical end-points extractable from medical charts or knowledge sources that characterize an individual patient's clinical and public health outcomes. A significant increase in our knowledge of the molecular epidemiology of biothreats has been reflected in the explosion of NCBI submissions (genomic data with or without annotation) and PubMed publications related to new variants of virulence or antibiotic resistance genes and their spread in hospitals, communities, countries and continents over the last five years (Figure 3).

**Figure 4:**
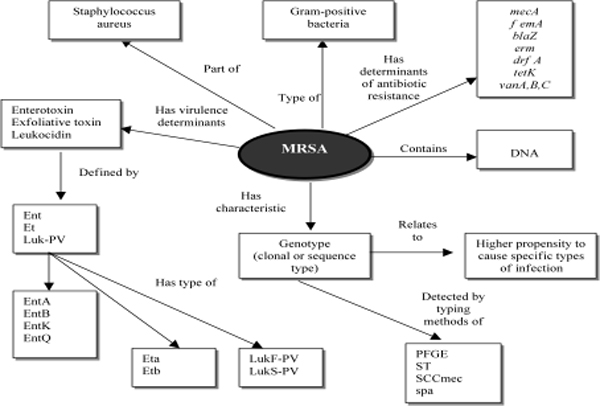
Relationships between the MRSA as a concept (object) and determinants of the pathogen profile. This data model defines major classes of attributes for MRSA profiles (e.g., genotyping methods, virulence factors and clinical outcomes) and relationships between them. *femA *= gene encoding a cytoplasmic protein necessary for the expression of methicillin resistance; *tst *– staphylococcal toxic shock toxin gene; *ent *– enterotoxin gene; *luk *= leukocidin gene; PV = Panton-Valentine leucocidin; *blaZ *= beta-lactamase gene; *mecA *= gene encoding PBP 2a, the low binding affinity penicillin binding protein that mediates methicillin resistance; *vanA, vanB, vanC *= vancomycin resistance genes; *erm *= macrolide resistance gene; *drfA *= trimethoprim resistance gene; *tetK *= tetracycline resistance gene; ST = sequence type; *SCCmec *= staphylococcus cassette chromosome *mec *gene type; *spa *= staphylococcal protein A gene type (Adapted from [3]).

Pathogen profiles can benefit from integration with knowledge representations that express the relationships and entities from the biological hierarchy. Ontologies and controlled vocabularies (UMLS, LOINC and SNOMED^®^) have emerged as tools for knowledge management [7,8]. Figure 4 presents the genomic profile of a hypothetical MRSA showing genomic markers of virulence, antibiotic resistance and clonality. Microbial profiles offer data models that are suited to semantic representations, which could be used both for reasoning as well as enhanced indexing. Vocabularies provide mechanisms for integrating high-level terms used in medical charts (e.g., "Tuberculosis" or "Food poisoning") with the low-level terms employed in the clinical bioinformatics (e.g., "MRSA SCCmec IV clone" or "*Salmonella typhimurium *MLVA 2-7-11-7-3 profile").

The majority of new genes and genomic markers are found in GenBank, while reports of the occurrence of known genes in new locations or their phenotypic characteristics are available from either Internet sources or peer-reviewed manuscripts. The presence or absence of particular genes or genetic elements can now be indicated by DNA or RNA sequencing-based typing systems which produce binary or octal representations. Information extraction will be an increasingly important measure for locating and assembling critical information relevant to the surveillance of infectious disease outbreaks in a timely fashion. There is already substantial prior work that aims to extract information from news articles reporting outbreaks, applied to online sources such as blogs and news feeds [9]. Text mining applications for detecting genetic mutations have also recently emerged [10]. In order to track the spread of pathogens and their various strains, we envision the mining of PubMed publications. Automated search agents and text mining utilities would be able to monitor new publications and check for say new strains of antibiotic resistance, tracking behavior over time.

### Biosurveillance pilot study

Near real time *Salmonella *surveillance was employed to test the framework and to further explore the potential of molecular typing-based early warning for outbreaks of diseases with epidemic potential. In early 2006, the Centre for Infectious Diseases & Microbiology, The University of Sydney, Westmead Hospital introduced routine *Salmonella typhimurium *(STM) fingerprinting. Specifically, clonally related strains were identified by multi-locus variable number tandem repeat analysis (MLVA) which was shown to be superior to gel-based typing for both surveillance and outbreak investigations of STM [12]. MLVA is based on the identification of short sequence repeats that vary in copy number in the microbial genome at various loci. MLVA detects polymorphisms at five different sites in the STM genome. Four regions of detection are on the bacterial chromosome and one is located on the serotype specific plasmid *pSLT*. MLVA has high discriminatory power within clonal species and appears to be more rapid and more amenable to standardization than pulse-field gel electrophoresis for both surveillance and outbreak investigations of STM [13].

**Figure 5:**
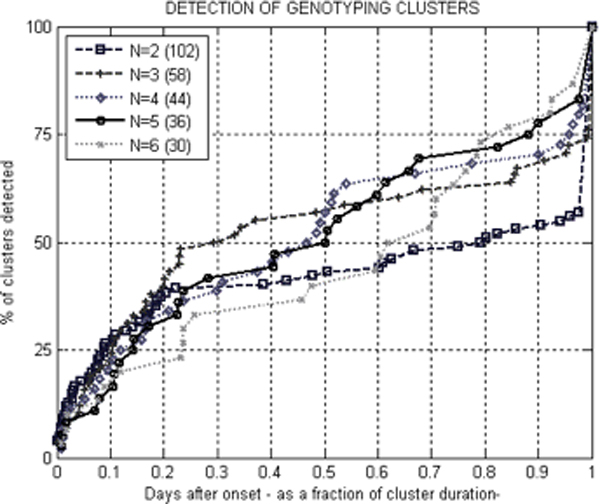
Impact of a cluster definition on the effectiveness of STM early warnings. Clusters defined as two (N = 2) or more isolates with the same MLVA genotype. Total numbers of isolates clustered are in parentheses.

**Figure 6:**
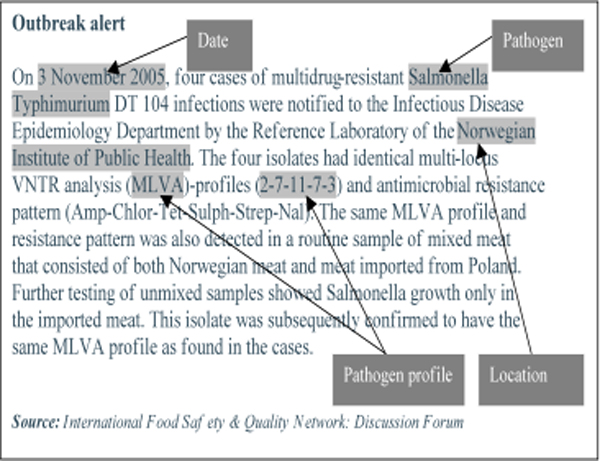
Example of Internet report on the STM cluster due to the specific MLVA profile which was identified in Norway with highlighted fields describing variables for text mining.

Prospective evaluation of rapid, molecular typing-based clustering of 816 STM isolates recovered from patients in New South Wales and Queensland in November 2006 – March 2007 identified 44 clusters involving 69% of all isolates tested. The average number of isolates per cluster was 12 (range 4–67). Three distinct temporal and spatial patterns were identified: (1) new point-source clusters localized in space and time, (20 geographically distributed cases of STM infections over a short period of time, and (3) the endemic activity of STM. An MLVA-based system demonstrated better discrimination power than traditional phage typing, identifying 9 and 11 independent clusters within each of two endemic phage types PT170 and PT135a, respectively. The performance of the detection system was significantly affected by the cluster definition employed. Around 50% of STM outbreaks are detected in the first half of their duration (Figure 5). This definition should take into account the local prevalence of STM clones as well as the public health resources available to investigate detected outbreaks. Descriptions of outbreaks caused by STM MLVA genotypes and implicated food sources have been also identified in publicly available Internet sources. Figure 6 provides an example of such a description with attributes employed in the text mining.

## Discussion

### Early warning for population health and infection control

Microbial genotyping improves the efficiency of outbreak investigations as it confirms or refutes epidemiological links among cases and between cases and potential environmental sources, thus triggering public health investigations. In our pilot, two types of early warning were considered: frequency alarm and the new MLVA type alarm. However, the utility of pathogen profiling goes beyond specific questions related to the investigation of possible outbreaks. It can be used also for disease monitoring, by identifying the transmission events and the associations between microbial types and clinical outcomes. Molecular profiling can assist in the assessment of the reproductive rates of infection during epidemics, in making infection control policies more pathogen-specific [3]. Molecular typing also facilitates the detection of chains and patterns of infection transmission and the construction of epidemic trees.

More bacterial genomes are being sequenced by the growing number of laboratories and genomic data stored and annotated online. Public databases such as MLSTNet , PulseNet  and the BioPortal , among others, allow access and matching of bacterial or viral isolates [14,15]. Linking systematically annotated profiles with clinical and research databases can identify previously unrecognized associations between phenotype, genotype, environment and host responses and can potentially identify the specific genes that govern them. Networks, which are created by relationships among phenotype, disease expression, environment, experimental context and associated genes with differential expression, could provide new insights into microbial interactions and pathogenesis [3,14]. This approach has been fruitful in metagenomics, and information management systems, capable of assisting with genotyping or functional genomics are being developed.

A particularly interesting prospect of this framework is the integration of molecular typing with epidemiological information, potentially achieving the global real-time epidemiological surveillance of pathogens with epidemic potential. Our MLVA pilot relied on a single database source for strain comparisons. However, surveillance typing networks such as SeqNet or second-generation PulseNet have improved the speed of outbreak detection and made possible the exchange of data between laboratories. In such 'on-line' surveillance systems, novel and previously characterized strains can be compared and grouped by cluster analysis. Spatial surveillance using emerging geographic information systems enhances the likelihood that even localized events will be detected and their extent and variables measured in space and time [16]. The output from these systems ultimately needs to be integrated into clinical and diagnostic processes.

The systematic construction of pathogen profiles would enable data to be integrated and shared, which is essential for successful surveillance and disease management. The heterogeneity of pathogen, host and environment imply that any individual marker is rarely sufficient to reflect the clinical and epidemiological complexity and ensure a reliable prediction of outcomes. However, a combination of markers is more likely to do so. Current genomic technology can rapidly and reliably produce this type of data from large numbers of strains to monitor routes of transmission or contribute to inventories of microbial gene polymorphisms and translate them into innovative typing procedures. Selective gene profiling can be directly linked to phenotypic characteristics such as virulence and antimicrobial resistance.

### Antimicrobial therapy optimization

Innovative bioinformatics tools that predict drug resistance or response to therapy from genotype have been developed to provide clinical decision support. These tools use either a statistical approach, in which the inferred model and prediction are treated as regression problems, or machine learning algorithms, in which the model is addressed as a classification problem. A statistical learning approach to the ranking of therapeutic choices often relies on a direct correlation between the baseline microbial profile, the therapeutic decision and the response to treatment e.g. expected reduction in viral load resulting from anti-HIV combination therapy [17]. It highlights the need for databases that can be widely shared, and allow the correlation of quality-controlled data from genotypic resistance assays and treatment regimens with short- and long-term clinical outcomes.

## Conclusion

The bioinformatics assisted infectious disease control framework targets questions of immediate public health and clinical utility and utilizes the molecular genotyping of biothreats in a clinically focused manner. The concept of pathogen profiling described here provides a framework for data integration and sharing to ensure that the flood of data from new molecular technologies will be used effectively in public health surveillance and disease management. The identification of new sets of molecular markers and the development of biosurveillance focused text mining tools will enable innovative **integrated infectious disease outbreak detection and response environments **based upon bioinformatics knowledge models and measured by outcomes including the accuracy and timeliness of outbreak detection and response.

## Methods

### Microbial profiling

Octal code profiles were assigned to each isolate for comparison and spatio-temporal clustering. A *genotyping cluster *was defined as a *maximal set *of at least four isolates that share the same MLVA genotype, among a set of isolates from infected patients, each with an associated date and location (e.g., collection date and patient's residential address) [13]. This definition was tested retrospectively using the space-time permutation scan statistic method with default values for bonds on cluster size (50% of population at risk and 50% of study period).

### System implementation

The system was implemented as both a stand-alone and as a web application with Map Objects (ESRI) GIS for map visualisation. MLVA patterns were matched using hierarchical cluster analysis. Minimum spanning trees analysis was also used to overcome the potential loss of information introduced by the latter.

## Competing interests

The authors declare that they have no competing interests.

## Authors' contributions

All authors contributed equally to this effort through discussion, writing, and revision of the manuscript.
